# Protein Hydrolysates from Salmon Heads and Cape Hake By-Products: Comparing Enzymatic Method with Subcritical Water Extraction on Bioactivity Properties

**DOI:** 10.3390/foods13152418

**Published:** 2024-07-30

**Authors:** Carla Pires, Matilde Leitão, Maria Sapatinha, Amparo Gonçalves, Helena Oliveira, Maria Leonor Nunes, Bárbara Teixeira, Rogério Mendes, Carolina Camacho, Manuela Machado, Manuela Pintado, Ana Rita Ribeiro, Elsa F. Vieira, Cristina Delerue-Matos, Helena Maria Lourenço, António Marques

**Affiliations:** 1 Division of Aquaculture, Upgrading and Bioprospection, Portuguese Institute for the Sea and Atmosphere (IPMA, I.P.), Av. Doutor Alfredo Magalhães Ramalho 6, 1495-165 Algés, Portugal; maria.sapatinha@ipma.pt (M.S.); amparo@ipma.pt (A.G.); helaoliveira@gmail.com (H.O.); barbara.p.b.teixeira@gmail.com (B.T.); rogerio@ipma.pt (R.M.); helena@ipma.pt (H.M.L.); amarques@ipma.pt (A.M.); 2 Interdisciplinary Centre of Marine and Environmental Research (CIIMAR/CIMAR-LA), University of Porto, Terminal de Cruzeiros do Porto de Leixões, Av. General Norton de Matos s/n, 4450-208 Matosinhos, Portugal; nunes.leonor@gmail.com (M.L.N.); carollcamacho@ipma.pt (C.C.); 3 Department of Chemistry, Nova School of Science and Technology, Nova University Lisbon, Campus da Caparica, 2829-516 Caparica, Portugal; 10440@eshte.pt; 4 Centre for Biotechnology and Fine Chemistry (CBQF), Universidade Católica Portuguesa, Rua de Diogo Botelho, 1327, 4169-005 Porto, Portugal; mmachado@ucp.pt (M.M.); mpintado@ucp.pt (M.P.); 5 Blue Bioeconomy CoLAB, Av. da Liberdade s/n, 4450-718 Leça da Palmeira, Portugal; aribeiro@b2e.pt; 6 Associated Laboratory for Green Chemistry (LAQV) of the Network of Chemistry and Technology (REQUIMTE), Instituto Superior de Engenharia do Porto, R. Dr. António Bernardino de Almeida 431, 4249-015 Porto, Portugal; elsavieiraf@gmail.com (E.F.V.); cmm@isep.ipp.pt (C.D.-M.)

**Keywords:** FPH, fish waste, enzymatic hydrolysis, SWH, biochemical composition, contaminants, cytotoxicity, antioxidant, ACE, α-amylase inhibitory activities

## Abstract

Fish by-products can be converted into high-value-added products like fish protein hydrolysates (FPHs), which have high nutritional value and are rich in bioactive peptides with health benefits. This study aims to characterise FPHs derived from salmon heads (HPSs) and Cape hake trimmings (HPHs) using Alcalase for enzymatic hydrolysis and Subcritical Water Hydrolysis (SWH) as an alternative method. All hydrolysates demonstrated high protein content (70.4–88.7%), with the degree of hydrolysis (DH) ranging from 10.7 to 36.4%. The peptide profile of FPHs indicated the breakdown of proteins into small peptides. HPSs showed higher levels of glycine and proline, while HPHs had higher concentrations of glutamic acid, leucine, threonine, and phenylalanine. Similar elemental profiles were observed in both HPHs and HPSs, and the levels of Cd, Pb, and Hg were well below the legislated limits. Hydrolysates do not have a negative effect on cell metabolism and contribute to cell growth. HPSs and HPHs exhibited high 2,2′–azino-bis(3 ethylbenzthiazoline-6)-sulfonic acid (ABTS) radical scavenging activity, Cu^2+^ and Fe^2+^ chelating activities, and angiotensin-converting enzyme (ACE) inhibitory activity, with HPHs generally displaying higher activities. The α-amylase inhibition of both FPHs was relatively low. These results indicate that HPHs are a promising natural source of nutritional compounds and bioactive peptides, making them potential candidates for use as an ingredient in new food products or nutraceuticals. SWH at 250 °C is a viable alternative to enzymatic methods for producing FPHs from salmon heads with high antioxidant and chelating properties.

## 1. Introduction

In 2021, total fish production reached approximately 182 million tonnes. Of this amount, 162 Mt (89%) were for human consumption, while the remaining 20 Mt (11%) were allocated for non-food uses [1]. Traditionally, 9% of this material is converted into fish meal and fish oil. However, the significant quantities of by-products from fish industries (including heads, frames, trimmings, skins, and bones), which can constitute 50–70% of the processed fish, can be converted into high-value-added products such as fish protein concentrates (FPCs) and fish protein hydrolysates (FPHs) [2]. FPHs offer nutritional, biological, and functional benefits. This sustainable approach not only reduces waste but also offers the opportunity to obtain bioactive peptides, which can be used as functional ingredients in food and nutraceutical products [2]. The bioactive peptides are made up of short amino acid sequences, which are inactive in the parent protein and can be released through enzymatic hydrolysis with commercially available enzymes such as Flavourzyme, Neutrase, and Alcalase [3]. Although the use of enzymes allows the prediction of which peptides can be generated, high-quality enzymes have a significant cost, which makes the enzymatic process expensive. This process is not rapid, and it can take hours to achieve the desired degree of hydrolysis. It also uses acids and bases to control the pH process, generating waste streams, and the process requires high energy consumption. Therefore, Subcritical Water Hydrolysis (SWH) can serve as an eco-friendly alternative to conventional hydrolysis methods. This technique can be employed to release bioactive peptides from various proteinaceous materials, including fish by-products. Subcritical water, also known as hot-compressed or pressurised hot water, is defined as water under conditions above its boiling point at 1 atm (>100 °C at 0.1 MPa) but below its critical point (374 °C at 22 MPa), with enough pressure to maintain its liquid state [4]. Under these conditions, the dielectric constant of water decreases with increasing temperature, allowing it to effectively dissolve moderately polar to non-polar substances. Additionally, the ionic product of water increases significantly, enhancing its ability to act as an acid or base catalyst, thus facilitating protein hydrolysis [4]. The use of subcritical water for protein hydrolysis has been explored with various fish by-products: SWH has been applied to bigeye tuna (*Thunnus obesus*) skin, abalone (*Haliotis discus hannai* Ino) viscera, cod (*Gadus morhua*) frames, and sardine (*Sardina pilchardus*) wastes [5,6,7,8].

FPHs from by-products are a promising source of bioactive peptides with antioxidant, antihypertensive, and anti-diabetic properties, among others [9,10]. Oxidative stress, caused by an imbalance between reactive oxygen species (ROS) production and the body’s antioxidant defences, contributes to chronic diseases like cardiovascular disorders, cancer, and neurodegenerative conditions. Antioxidant peptides from FPHs can combat oxidative damage by scavenging free radicals and inhibiting lipid peroxidation. Studies have shown the antioxidant potential of FPHs from various fish by-products, including heads, bones, frames, skins, muscles, and viscera [3,10,11,12,13,14].

FPHs also exhibit significant antihypertensive effects, with peptides acting as angiotensin-converting enzyme (ACE) inhibitors, which help regulate blood pressure by modulating the renin–angiotensin–aldosterone system. Research has demonstrated the ACE-inhibitory potential of FPHs from muscles, heads, bones, and other parts like blood and roe [3,15,16]. Furthermore, FPHs have shown the ability to inhibit α-amylase and α-glucosidase, enzymes involved in carbohydrate digestion, thereby helping manage blood sugar levels in diabetes [3,17,18].

Portugal imports large amounts of salmon and Cape hake for further production of fish portions and fillets. The by-products generated in these processes are readily available and represent a high-quality raw material (good protein and low microbiological contamination). Therefore, this study aims to explore the bioactive potential of enzymatic fish protein hydrolysates (FPHs) derived from salmon heads and hake by-products using Alcalase, as well as hydrolysates obtained through subcritical water hydrolysis (SWH) from salmon heads (see Figure S1 in the Supplementary Material). The nutritional composition, amino acid and mineral profiles, chemical contaminants (Pb, Cd, and Hg), bioactivities (antioxidant, ACE inhibitory, and α-amylase inhibitory activities), and cytotoxicity and impact on Caco-2 cell proliferation were analysed in the enzymatic hydrolysates. Additionally, the antioxidant activities of the salmon heads hydrolysates prepared by SWH were evaluated.

## 2. Materials and Methods

### 2.1. Fish Material

Fresh heads of farmed Atlantic salmon (*Salmo salar*) were purchased at a local supermarket and transported to the laboratory on ice. Additionally, trimmings (muscular fraction and bones from the head and tail) of Cape hake (*Merluccius capensis*), resulting from the frozen hake filleting processing, were also used. These by-products were kindly provided by Gelpeixe, Alimentos Congelados S.A. (Loures, Portugal). Both raw materials were ground and minced in an industrial meat grinder (HOBART, Troy, OH, USA) and stored at −80 °C until further use.

### 2.2. Alcalase Hydrolysis of Salmon Heads and Hake By-Products

Freeze-grinded raw materials were thawed and used in the preparation of the FPHs according to the method described by Henriques et al. (2021), with slight modifications. One kilogram of freeze-grinded raw material was homogenised with 2 L of distilled water (1:2 *w*/*v*), and the mixture was incubated at 60 °C in a 5 L glass reactor under agitation. The pH of the mixture was adjusted to 8.5, and the hydrolysis process was initiated by adding 1% (*w*/*w*) of Alcalase. The pH was maintained at 8.5 during the hydrolysis time (3 h) with the addition of 2 M NaOH. Then, the reaction was stopped by increasing the temperature to 90 °C. After 20 min at 90 °C, the mixture was cooled at room temperature and centrifuged at 4 °C, 10,000× *g*, for 20 min. The supernatant was filtered, freeze-dried and stored at −80 °C until further analysis. The hydrolysate prepared from the salmon heads was designated as HPS, and the one prepared from the Cape hake trimmings was designated as HPH [3].

### 2.3. Subcritical Water Hydrolysis of Salmon Heads

The preparation of FHPs from salmon heads by SWH was carried out according to the method described by Nilsuwan et al. (2022) [19]. The lipid fraction was previously removed by adding isopropanol (C_3_H_8_O) at 30% (*v*/*v*) in a ratio of 1:10 (*m*/*v*) to the minced salmon heads. The mixture was homogenised with a magnetic stirrer at 150 rpm for 60 min at refrigeration temperature (4 °C). The deposited mass was collected and washed with distilled water (1:10 *w*/*v*). The washing process was repeated three times, and finally, the sample was drained. The attained moisture content of the sample was 63.3 ± 1.3%. The hydrolysis process with subcritical water was carried out in a Parr Series 450 reactor (Moline, IL, USA) with a Parr 4848 controller. Extractions were carried out using approximately 2 g of sample and 200 mL of distilled water at 300 rpm, testing different combinations of temperature (200 or 250 °C), pressure (50 or 100 bar) and extraction time (10 or 30 min), as described in Table S1 (in Supplementary Material). After centrifugation, the supernatant was freeze-dried and stored at −80 °C until use. The hydrolysates were designated as SWH1, SWH2, SWH3, SWH4, SWH5, and SWH6.

Although −80 °C is not an industrial temperature, it was used in this study to store ground raw materials and the FPHs until further analysis. This temperature was selected to prevent biochemical changes, such as oxidation, and because the preparation and characterisation of different FPH batches were carried out at different times, this procedure ensured that no additional variables were introduced.

### 2.4. Hydrolysis and Protein Yields

The hydrolysis and protein yields of the different processes were determined according to the following [20,21]:

(1) $$Hydrolysis\;yield\left(\%\right)=\frac{W_{f}}{W_{i}}\times 100,$$

(2) $$Protein\;yield\left(\%\right)=\frac{P_{f}}{P_{i}}\times 100,$$

where W_f_ is the weight (in grams, dw) of the FPH, W_i_ is the weight (in grams, dw) of raw material, P_f_ is the total protein content (in grams) of the FPH, and P_i_ is the total protein content (in grams) of raw material.

### 2.5. Degree of Hydrolysis

The degree of hydrolysis (DH) is defined as the percentage of the number of peptide bonds cleaved compared to the total number of peptide bonds in the substrate during the enzymatic reaction. The DH was determined using the o-phthalaldehyde (OPA) method described by Nielsen et al. (2001) based on the measurement of the amino groups generated during the hydrolysis process [22]. A solution of FPH was added to 3 mL of the OPA solution and mixed for 5 s. The mixture stood for exactly 2 min before reading the absorbance at 340 nm in an Evolution 201 UV-Visible Spectrophotometer (Thermo Scientific, Waltham, MA, USA). The blank was prepared using the same volume of distilled water instead of a hydrolysate sample. A serine solution with a concentration of 0.9516 meq/L was used as a reference. The degree of hydrolysis was calculated according to the following equation:

(3) $$DH\left(\%\right)=\left[\frac{{Abs}_{sample}-{Abs}_{blank}}{{Abs}_{serine}-{Abs}_{blank}}\times \frac{\left(0.9516\times 10\right)}{W\times N\times 6.25}\right]\times \frac{100}{8.6},$$

where Abs_sample_ is the absorbance of FPH solution, Abs_blank_ is the absorbance of blank, Abs_serine_ is the absorbance of serine solution, W is the weight in grams of the FPH, and N is the total nitrogen (%) in the FPH.

### 2.6. Proximate Composition of Fish Raw Material and FPHs

The ash, fat and moisture contents of raw material and FPHs were determined as described in AOAC (2005) [23]. The protein content was determined using an FP-528 LECO nitrogen analyser (LECO, St Joseph, MI, USA) calibrated with ethylenediaminetetraacetic acid (nitrogen = 9.57 ± 0.03%), following the Dumas method as described by Saint-Denis & Goupy (2004) [24]. All measurements were performed in triplicate. The results of fish raw material were expressed as % of wet weight (ww), while for FPHs, the results were expressed as % of dry weight (dw).

### 2.7. Amino Acid Compositions

The quantification of amino acids in raw materials and FPHs was performed using the hydrolysis method described in AOAC (2005) [23]. For total amino acid extraction, ca. 15–35 mg of freeze-dried sample (1.5–2.0 mg of nitrogen) were acid hydrolysed with 3 mL of 6 M HCl (Merck, Kenilworth, NJ, USA) containing 0.1% phenol (Merck, Kenilworth, NJ, USA). Norvaline (99%, Sigma-Aldrich Corp., St. Louis, MO, USA) and sarcosine (98%, Sigma-Aldrich Corp., St. Louis, MO, USA) were further added to samples (final concentration of 500 pmol/μL) before hydrolysis and used as internal standards. Hydrolysis tubes were vacuumed, fluxed, and capped under a nitrogen atmosphere, and the samples were hydrolysed at 110–115 °C for 24 h. After hydrolysis, samples were neutralised with 6 M NaOH, quantitatively transferred into 20 mL volumetric flasks with ultrapure water, filtered using cellulose membrane syringe filters (0.2 µm pore size) and stored at −80 °C until analysis. The chromatographic conditions used were in accordance with the Agilent method described by Henderson et al. (2000), and amino acid separation was performed using high-performance liquid chromatography (Agilent 1100 HPLC, Agilent Technologies, Palo Alto, CA, USA) in a Phenomenex Gemini ODS C18 guard column (4 × 3 mm), and a Phenomenex Gemini ODS C18 110 Å column (4.6 × 150 mm, 5 μm) (Phenomenex Inc., Torrence, CA, USA) using pre-column derivatisation with OPA and 3-mercaptopropionic acid in borate buffer (Agilent Technologies, Palo alto, CA, USA) and 9-fluorenylmethyl chloroformate in acetonitrile (FMOC, Agilent Technologies, Palo Alto, CA, USA) [25]. Detection was performed by fluorescence analysis (340/450 nm and 266/305 nm). Identification and quantification of amino acids were evaluated by comparing their retention times and peak areas with those of standard amino acids (Sigma, MO, USA) ranging from 9 to 900 pmol/μL using Agilent ChemStation software Rev.A.10.02 (1757) for LC (Agilent Technologies, Palo Alto, CA, USA). All measurements were conducted in duplicate. It should be noted that cysteine and tryptophan might undergo degradation during acid hydrolysis.

Amino acids scores for hake and salmon hydrolysates were calculated based on the reference requirements for adults (FAO/WHO/UNU, 2007) as follows:

(4) $$Amino\;acid\;score\left(\%\right)=\frac{mg\;of\;amino\;acid\;per\;g\;of\;test\;protein}{mg\;of\;amino\;acid\;requirement*}\times 100$$

* FAO/WHO/UNU, 2007

### 2.8. Mineral Profile and Contaminants Metals

The elements chromium (Cr), copper (Cu), iron (Fe), magnesium (Mg), manganese (Mn), nickel (Ni), potassium (K), sodium (Na), and zinc (Zn) were measured by flame atomic absorption spectrophotometry (Spectr AA 55B, Varian, Palo Alto, CA, USA) with a background deuterium correction, according to official analytical methods [26]. Concentrations were determined through linear calibration obtained from absorbance measurements of at least five different concentrations of standard solutions: Cr(NO_3_)_2_, Cu(NO_3_)_2_, Fe(NO_3_)_3_, Mg(NO_3_)_2_, Mn(NO_3_)_2_, KNO_3_, NaNO_3_, Ni(NO_3_)_2_, and Zn(NO_3_)_2_ (1 g/L dissolved in 0.5 M HNO_3_). Detection limits for the elements were 0.09 (Cr), 0.02 (Cu), 0.3 (Fe), 0.02 (Mg), 0.01 (Mn), 0.01 (K), 0.09 (Na), 0.02 (Ni), and 0.06 (Zn) mg/kg.

Cadmium (Cd) and lead (Pb) were determined using graphite furnace atomic absorption spectrometry, using a Varian apparatus Spectr 220Z with a Zeeman correction. The methodology followed was based on the European Standard EN 14084 [27]. The concentrations were determined through linear calibration obtained from absorbance measurements of at least five different concentrations of standard solutions: Pb(NO_3_)_2_ and Cd(NO_3_)_2_ (1 g/L in 0.5 M HNO_3_). The quantification of total mercury (HgT) was performed using atomic absorption spectrophotometry using a direct mercury analyser in a mercury analyser spectrophotometer (AMA 254, Leco, St. Joseph, MI, USA) [28]. Detection limits for the three metals were 0.002 (Cd), 0.004 (Hg), and 0.02 (Pb) mg/kg.

All analyses were performed in duplicate and analytical data was reported as mg/kg of products on a dry weight basis. Two certified reference materials were tested in the same conditions as the samples in order to assess analytical method accuracy: LUTS-1 (Non-defatted lobster hepatopancreas reference material for trace metals) and DORM-5 (Fish Protein Certified Reference Material) from the National Research Council of Canada. The obtained values for all elements were in good agreement with the certified values.

### 2.9. Molecular Weight (MW) of FPHs

The peptide profile of HPSs and HPHs was determined by size exclusion chromatography (SEC) in an FPLC ÄKTA system (Amersham Biosciences, Uppsala, Sweden) using a Superdex Peptide 10/300 GL column with a UV detector at 254 nm and 280 nm [20]. The eluent was acetonitrile/water (30/70) and 0.1% trifluoroacetic acid, and the flow rate was 0.5 mL/min. The approximate molecular weights were estimated using a molecular weight calibration curve prepared with ribonuclease A (13,700 Da), aprotinin (6500 Da), angiotensin I (1296 Da), triglycine (189 Da), and glycine (78 Da). The percentage of the molecular weight of the peptides was calculated by dividing the area of the identified peak by the total area of all peaks.

### 2.10. Cytotoxicity and Proliferation of FPHs

#### 2.10.1. Cell Line Growth Conditions

Human Caucasian colon carcinoma epithelial cells, Caco-2 (ECACC 86010202 European Collection of Authenticated Cell Cultures), cells were grown in Dulbecco’s modified Eagle’s medium (Lonza, Basel, Switzerland) supplemented with 10% (*v*/*v*) heat-inactivated foetal bovine serum (Biowest, France), 1% (*v*/*v*) Penicillin–Streptomycin–Fungizone (Lonza, Basel, Switzerland), and 1% (*v*/*v*) non-essential amino acids (Lonza, Basel, Switzerland). Cells were incubated at 37 °C in a humidified atmosphere with 5% CO_2_.

#### 2.10.2. Cytotoxicity

Cytotoxicity evaluation was performed according to the ISO 10993-5:2009 standard [29]. Cells were grown to 80–90% confluence, detached using TrypLE Express (ThermoScientific, Waltham, MA, USA), seeded at 1 × 10^4^ cells/well in 96-well tissue cultured plates (Nunclon ThermoScientific, Waltham, MA, USA), and allowed to adhere for 24 h. Afterwards, the media was carefully removed and replaced with media supplemented with sterile filtered (0.22 µm) fish hydrolysates at concentrations between 0.3 and 20 mg/mL. Dimethyl sulfoxide (DMSO) at 40% in culture media was used as a death control, and plain culture media was used as a growth control. After the incubation time, Presto Blue (ThermoFisher, Waltham, MA, USA) was added to each well and incubated at 37 °C in the darkness for 1 h. After this period, fluorescence (Excitation: 560 nm; Emission: 590 nm) was measured using a microplate reader (Synergy H1, Biotek Instruments, Winooski, VT, USA). All assays were performed in quadruplicate.

#### 2.10.3. Cell Proliferation

To evaluate the sample’s impact upon cellular proliferation, the cells were grown to 80–90% confluence, detached using TrypLE Express (ThermoScientific, MA, USA), and seeded at 1 × 10^4^ cells/well in a 96-well microplate (Nunclon Delta, ThermoScientific, MA, USA). After 24 h, the culture media was carefully removed and replaced with a culture media supplemented with samples at the selected concentration. DMSO (Sigma, St. Louis, USA) at 10% (*v*/*v*) in culture media was used as a non-proliferation control, and plain culture media was used as a proliferation control. After 24 h of incubation, to evaluate cellular proliferation, Cyquant Direct Cell Proliferation (ThermoScientific, MA, USA) was used according to the manufacturer’s instructions, with fluorescence (Ex: 480 nm; Em: 535 nm) being measured using a microplate reader (Synergy H1, Biotek Instruments, Winooski, VT, USA). All assays were performed in quadruplicate.

### 2.11. Antioxidant Activity

#### 2.11.1. 2,2-Diphenyl-1-picrylhydrazyl (DPPH) Radical Scavenging Activity

The determination of DPPH radical scavenging activity was performed according to the method of Shimada et al. (1992) with adjustments [3,30]. All analyses were made at least in triplicate and the results are presented as mean values. The EC_50_ value was calculated for each hydrolysate prepared by the conventional methodology. In the case of salmon protein hydrolysates prepared by SWH, the concentration tested was 0.1 mg/mL.

#### 2.11.2. 2,2′-Azino-bis(3 ethylbenzthiazoline-6)-sulfonic Acid (ABTS) Radical Scavenging Activity

The ABTS radical scavenging activity of FPHs was performed according to Re et al. (1999) and as described by Henriques et al. (2021) [3,31]. All determinations were made at least in triplicate and the EC_50_ value was calculated for each hydrolysate prepared by the conventional methodology. In the case of salmon protein hydrolysates prepared by SWH, the concentration tested was 0.1 mg/mL.

#### 2.11.3. Reducing Power (RP)

The reducing power was determined following Oyaizu’s method (1988) described by Henriques et al. (2021) [3,32]. All analyses were carried out at least in triplicate. The concentration for the absorbance value of 0.5 value (A_0.5_) was determined for each hydrolysate prepared by the conventional methodology. In the case of salmon protein hydrolysates prepared by SWH, the concentration tested was 0.1 mg/mL.

### 2.12. Metal Chelating Activities

#### 2.12.1. Cu^2+^ Chelating Activity

The copper chelating activity was evaluated by copper chelate titration using pyrocatechol violet (PV) as the metal chelating indicator as described by Torres-Fuentes et al. (2011) with slight modifications [3,33]. All determinations were carried out at least in quadruplicate, and the EC_50_ was estimated for each hydrolysate prepared using the conventional methodology. In the case of salmon protein hydrolysates prepared by SWH, the concentration tested was 0.1 mg/mL.

#### 2.12.2. Fe^2+^ Chelation Activity

The iron chelating activity of the FPH was estimated using the method described by Decker and Welch (1990), with modifications according to Henriques et al. (2021) [3,34]. All determinations were carried out at least in quadruplicate, and the EC_50_ value was determined for each hydrolysate prepared using the conventional methodology. In the case of salmon protein hydrolysates prepared by SWH, the concentration tested was 0.1 mg/mL.

### 2.13. ACE Inhibitory Activity

The ACE inhibitory activity using hippuryl-L-histidyl-L-leucine (HHL) as the substrate was evaluated using high-performance liquid chromatography (HPLC) according to the methodology described by Pires et al. (2015) [35]. Captopril was used as a commercial inhibitor and showed an ACE inhibitory activity of ca. 95%.

### 2.14. α-Amylase Inhibitory Activity

The α-amylase inhibitory activity was measured using the method described by Hansawasdi et al. (2000) [36]. All the assays were performed at least in triplicate, and the results are presented as mean values.

### 2.15. Statistical Analysis

The results of the analyses are reported as mean values ± standard deviation (SD). All statistical analyses were performed using the STATISTICA^©^ software version 12 from StatSoft, Inc. (Tulsa, OK, USA). All data were checked for normality of distribution and homogeneity of variances using Shapiro–Wilk and Levene’s tests, respectively. Differences between the mean values of two groups were tested using a Student’s t-test. Differences among mean values of the groups were tested using a one-way analysis of variance (ANOVA), followed by Tukey’s HSD multiple comparison test. Pearson correlation was also performed between several variables (e.g., amino acids, DH, MW, DPPH and ABTS radical scavenging activities, RP, Fe^2+^ and Cu^2+^ chelating activities, and ACE and α-amylase inhibitory activities). Statistical significance was considered at *p* < 0.05.

## 3. Results and Discussion

### 3.1. Proximate Composition, Degree of Hydrolysis, and Protein Yield

Table 1 summarises the proximate composition of raw salmon heads and hake by-products, as well as their corresponding Alcalase protein hydrolysates. Significant differences (*p* < 0.05) in fat and moisture contents were observed between hake by-products and salmon heads, primarily attributable to the notably high-fat content of the latter (19.8% on a wet weight basis). The respective hydrolysates (HPSs and HPHs) presented high levels of protein (ca 76% dw) and moisture and fat contents lower than 7.6% dw and 2.7% dw, respectively. Several authors have reported similar protein and moisture contents for FPHs prepared from different by-products [2,3,37]. It is noteworthy that the relatively low-fat content obtained in the HPS is due to the removal of lipids released in the hydrolytic process (ca 95%). Most of the studies reported FPHs with a fat content lower than 5% dw, while only a small number mentioned FPHs with fat content exceeding 5% dw [2,3,37]. The relatively high ash content of HPSs and HPHs is due to the addition of NaOH for pH adjustment during the hydrolysis process. The reported ash content for FPHs prepared from different fish by-products ranged between 0.45 and 25.94% dw [2,3,37]. Indeed, ash content depends on the hydrolysis process and the type of by-products used.

The hydrolysis protein yield can vary depending on the type of by-product, enzymatic conditions, and the specific enzyme employed. As shown in Table 1, the protein yield obtained in the preparation of HPSs was significantly lower (*p* < 0.05) than that obtained in the preparation of HPHs (75.5%). Likely, the higher fat percentage in salmon heads leads to protein loss through emulsion formation with the fat, resulting in a lower protein yield. However, the hydrolysis yield, based on the ratio of dried FPHs to the dried weight of raw material, obtained for HPSs (32.4%) was considerably lower compared to 76.4% for HPHs. These results highlight the high proportion of skin, bones, and fat in the salmon heads.

Gbogouri et al. (2004) reported a slightly higher protein yield (71.0%) for protein hydrolysates prepared from salmon heads with Alcalase [38]. However, the protein yield of hydrolysates prepared from salmon heads and backbones (64.7%) was relatively lower than that of HPSs [39]. According to Ramakrishnan et al. (2024), the protein yield for salmon protein hydrolysates prepared by different hydrolysis conditions (pH, time, temperature, and enzyme) and from different parts of salmon varied between 8.5 and 89.3% [37].

The yield obtained in the preparation of HPHs was relatively higher compared to the previously achieved in our laboratory for the preparation of FPHs from the same raw material using the same enzyme (70.5%) but lower than yields reported for FPHs prepared with Protamex (50.9%) [20,35]. Iñarra et al. (2023) reported protein yields ranging between 40 and 65% for protein hydrolysates prepared from hake by-products with different enzymes [40].

The DH results (Table 1) show that HPSs exhibited a higher DH (26.2%) compared to HPHs (20.3%). The DH of HPHs was relatively lower than that achieved in FPHs prepared from the same species using Alcalase (22.6%) [20]. This difference could be related to the type of material used in the preparation of FPHs. However, the DH achieved for HPHs and HPSs falls within the range reported by other authors for FPHs prepared from fish muscle, fish discards, and by-products with Alcalase [3,10,41].

### 3.2. Amino Acid Compositions

As can be seen in Table 2, the total amino acids determined in salmon heads (33.77 g/100 g) was lower than in the hake by-products (74.38 g/100 g), due to the lower proportion of protein (in a dry weight basis) in the salmon raw material. Considering such differences, the comparison among raw materials and hydrolysates was based on the amino acid contents expressed per 100 g of protein.

Regarding hake, the hydrolysis process with Alcalase resulted in the loss of several amino acids by at least 5%, including glutamic acid, aspartic acid, leucine, valine, isoleucine, phenylalanine, tyrosine, methionine, and histidine. In contrast, in salmon, amino acid loss was observed only for lysine, threonine, tyrosine, and histidine. This suggests that hydrolysis was more incomplete in the case of hake. In comparison, earlier research also noted a decline in the levels of several amino acids (such as lysine, methionine, proline, phenylalanine, isoleucine, tyrosine, and valine) in hake hydrolysates when using Protamex, whereas Gbogouri et al. (2004) observed a decrease solely in glycine and lysine following the hydrolysis of salmon heads with Alcalase [35,38]. Regarding salmon skin hydrolysates, both Alcalase and Protamex yielded similar amino acid profiles, except for proline, which exhibited a higher content in the use of the former enzyme, and methionine, which showed a higher content when prepared with the latter enzyme [42].

Salmon head-derived hydrolysates exhibited elevated levels of glycine (9.66 g/100 g of protein), proline (5.17 g/100 g of protein), and hydroxyproline (1.32 g/100 g of protein) compared to hake hydrolysates. These heightened concentrations of these amino acids may suggest a greater presence of collagen, sourced from the skin and bones, in salmon as opposed to hake raw materials. In a prior study, glycine (approximately 20–22 g/100 g, depending on the enzyme) was identified as the most abundant amino acid in hydrolysates prepared from salmon (*Salmo salar*) skin [42]. Additionally, significantly higher levels of glycine were detected in hydrolysates derived from salmon (*Salmo salar*) skin gelatin (approximately 23 g/100 g) compared to salmon trimmings (approximately 7 g/100 g) [43]. On the other hand, hake hydrolysates contained higher concentrations of glutamic acid, leucine, threonine, and phenylalanine than salmon head-derived hydrolysates.

From a nutritional standpoint, the amino acids scores (See Table S2 in Supplementary Material) for threonine, cysteine, lysine, and phenylalanine + tyrosine exceeded 150% among essential amino acids in both hydrolysates. On the other hand, only histidine exhibited a nutritional score below 100%, with values of 64.9% for HPHs and 93.4% for HPSs. However, when incorporating hydrolysates into new food product formulations, it is essential to consider evidence of tolerable upper intake levels for amino acids, as summarised by Elango (2023) [44]. This is especially critical for lysine, given its significant contribution to hydrolysates, alongside the recommended dietary allowance of 2.7 g/day and the lowest-observed-adverse-effect level of 7.5 g/day [45].

### 3.3. Mineral and Chemical Contaminants

The mineral profile of hake by-products, salmon heads, and protein hydrolysates is presented in Table 3. Similar elemental patterns were observed in both hake by-products and salmon heads and their hydrolysates. The mineral profile of raw materials showed a predominance of K > Na > Mg > Fe > Zn > Mn > Cu > Cr > Ni. For FPHs, the mineral elements were ordered as follows: Na, K, Mg, Fe, Zn, Cu, Ni, Mn, and Cr. These profiles were typical for seafood products [46]. As for macroelements, the highest K content was obtained in hake by-products, being statistically different from that obtained in salmon heads. In the case of Na and Mg, the values were not statistically different. However, in comparison with the respective hydrolysates (HPHs; HPSs), there was a significant decrease in Mg and a significant increase in Na content. This increase in Na can be explained by the introduction of NaOH during the hydrolysis process. Previous studies have reported higher levels of Mg in salmon heads and cod backbone hydrolysates compared to the findings of this study [47,48]. Regarding trace elements, salmon heads have higher values of Fe, Zn, and Mn compared to hake by-products. This may be mainly due to the presence of gill tissue in salmon heads [49]. In general, hydrolysates from hake by-products and salmon heads exhibited significantly lower levels of these three metals compared to the raw material, except for Fe. Iron and Zn values found in the present study were lower than those found in the study carried out by de la Fuente et al. (2023) [47]. The Cu content in salmon heads was higher than that in hake by-products, likely due to the presence of gill tissue. However, the Cu levels in the HPS were lower than in the raw material and statistically identical to those found in hake by-products. In relation to Cr and Ni, quantified contents were the usual for fish products; however, for Ni, no statistical differences were observed between the raw material and the respective hydrolysate. In the case of Cr, this element was not detected in the hydrolysates [49].

The contribution of each element, in percentage, was also calculated in relation to the Dietary Reference Intake (DRI) (Table 3) using the estimated daily intake for each element (average content, mg/kg) obtained for each hydrolysate considering one hundred grams [50,51]. The results indicate that the Na content in 100 g of FPH significantly exceeded the maximum recommended daily intake. However, by optimising the hydrolysate production process or incorporating an additional desalination step, the Na levels in the hydrolysates can be significantly reduced to meet the DRI value. Regarding the K levels, both hydrolysate samples were good sources of K (DRI: 26–33%), contributing a substantial portion of the essential mineral required. For Cu, Fe, and Zn, the hydrolysates contributed less than 10% of the daily recommended intake (DRI). Therefore, these FPHs can be considered beneficial dietary supplements or ingredients that enhance the nutritional value of food products.

Since the levels of contaminant metals can affect the use of HP, for example, as a food ingredient, the concentrations of Cd, Hg, and Pb were also evaluated (Table 3). The levels obtained for Cd and Pb in both raw materials and hydrolysates were almost always lower than the detection and quantification limits of the methods and lower than the limits legislated [52]. These results indicate low contamination in these products by these two metals. Identical levels were found by de la Fuente et al. (2023) [47]. As for Hg, the highest concentrations were obtained for hake by-products but still below the limit of 0.50 mg/kg (ww) indicated in the European Union Regulation (2023) [52]. In hake by-products, a decrease in the corresponding hydrolysates was observed due to Hg removal during the hydrolysis process. It should be noted that the values obtained in this study for salmon heads are similar to those obtained by de la Fuente et al. (2023) [47].

### 3.4. Molecular Weight Distribution of FPHs

**Figure 1:**
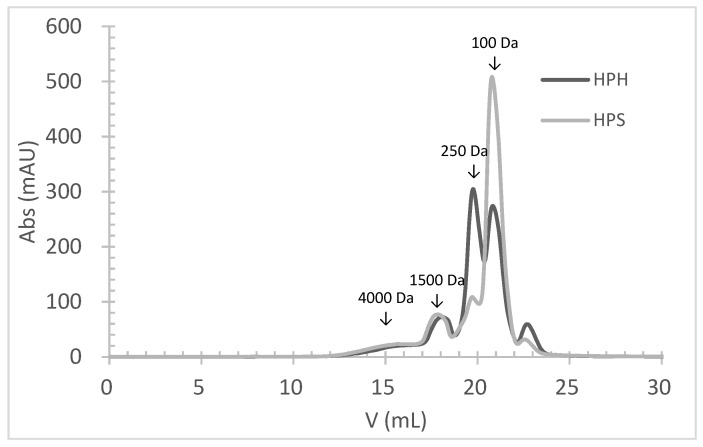
The molecular weight distribution of peptides obtained by size-exclusion chromatography analysis of protein hydrolysates prepared by Alcalase hydrolysis of hake by-products (HPHs) and salmon heads (HPSs).

The molecular weight (MW) distribution of HPHs and HPSs, obtained by size-exclusion chromatography, clearly demonstrates the hydrolysis of salmon and hake proteins into smaller MW peptides (Figure 1). The profile of HPHs and HPSs was very similar, and both hydrolysates had approximately 93% peptides with an MW below 1500 Da. The primary difference between HPH and HPS profiles was the percentage of peptides with an average MW of 250 Da and 100 Da. HPHs contained about 36% of peptides with an average MW of 250 Da, compared to 14% in HPSs. Conversely, HPHs had about 37% of peptides with an average MW of 100 Da, while HPSs had 62%. These findings align with the achieved DH, as HPSs exhibited a significantly higher (*p* < 0.05) DH value (26.2%) than HPHs (20.3%). This indicates that hydrolysates obtained from salmon heads underwent a higher degree of hydrolysis.

It is well known that a higher DH results in a higher percentage of lower MW peptides. In this study, a strong negative correlation was observed between the percentage of peptides with an MW above 1000 Da (MW > 1000 Da) and the DH (r = 0.962). Conversely, a positive correlation was found between the percentage of peptides with an MW below 1000 Da (MW < 1000 Da) and the DH (r = 0.770).

### 3.5. Cytotoxicity and Proliferation of FPHs

**Figure 2:**
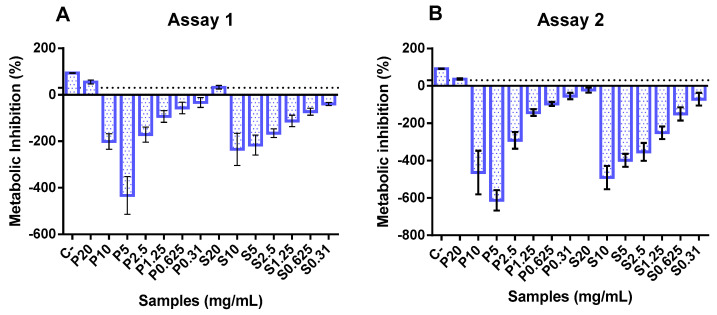
Cytotoxicity of protein hydrolysates prepared from hake by-products (P) and salmon heads (S) at different concentrations (0.31, 0.6, 1.25, 2.5, 5, and 10 mg/mL). The dotted line represents the 30% cytotoxicity limit. C−: control.

In the two independent assays, only the highest concentration (20 mg/mL) exerted an inhibitory effect upon Caco-2 cell metabolism, as can be seen in Figure 2. Conversely, concentrations between 0.3 and 10 mg/mL showed an apparent metabolic stimulation (negative values for metabolism inhibition), indicating a metabolic activity higher than that of the growth control. These data align with previous studies on fish hydrolysates, demonstrating that increasing the concentration of hydrolysates is safe and does not compromise cell metabolism. In the available literature, fish hydrolysates are usually tested at lower concentrations (below 1 mg/mL) [53,54,55].

**Figure 3:**
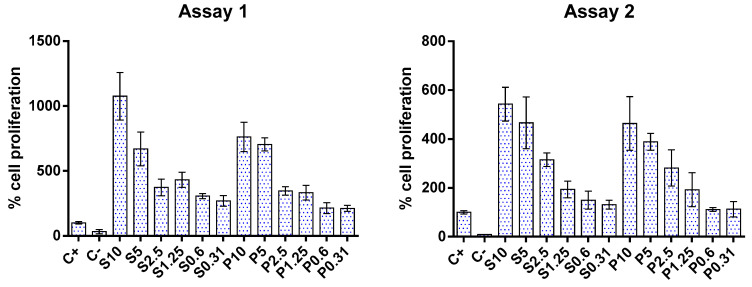
Impact of protein hydrolysates prepared from hake by-products (P) and salmon heads (S) at different concentrations (0.31, 0.6, 1.25, 2.5, 5, and 10 mg/mL) upon Caco-2 cell proliferation. C+: positive control; C−: negative control.

Regarding cell proliferation, the results of two independent assays showed that both fish hydrolysates promoted cell growth, as can be seen in Figure 3. This effect appears to be dose-dependent: as the concentration of hydrolysate decreased, cell proliferation also decreased. This finding corroborates the apparent increase in cell metabolism observed in the metabolic inhibition assay mentioned above. The increase in cell proliferation aligns with those reported in the literature for gut cells [54,56,57].

### 3.6. Antioxidant Activity

Measuring antioxidant capacity with a single method cannot accurately determine a compound’s overall ability to function as an antioxidant [58]. Hence, this study involved different methods, such as DPPH radical scavenging activity, ABTS radical scavenging activity, reducing power, and metal chelating activity (Cu^2+^ and Fe^2+^).

The DPPH radical scavenging capacity of HPHs and HPSs is present in Table 4. Both hydrolysates exhibited a DPPH scavenging activity with a concentration dependence, and this activity was significantly higher in the HPHs (EC_50_ = 9.5 mg/mL). The inhibition percentage values obtained in the current work (ca. 20%) were in the range (0.76–31.9%) of those reported for hydrolysates prepared from fish by-products (heads skins, bones, and whole fish) with Alcalase for a 3 mg/mL hydrolysate concentration [3]. However, the DPPH radical scavenging activity of HPHs and HPSs was double of that found in FPHs prepared from hake by-products [20]. Fish protein hydrolysates prepared from salmonid waste (heads, trimmings, and frames) and heads and viscera of monkfish using Alcalase demonstrated DPPH inhibition percentages ranging from 48.2% to 56.9% [39,59]. In contrast, protein hydrolysates from rainbow trout skins and from four Australian fish species exhibited significantly lower DPPH inhibition compared to the results of this study [60,61,62].

The DPPH radical scavenging activity did not align with the peptide profile of FPHs (Figure 1), as lower MW peptides are generally reported to exhibit higher DPPH activity [3,63]. However, other authors have found that hydrolysates with low DH (5%) exhibited higher DPPH radical scavenging activity than those with high DH (25%) [64]. The higher levels of methionine, phenylalanine, lysine, leucine, and tyrosine in the HPH might have contributed to the increased DPPH activity, as noted by Ryu et al. (2021) [10]. This author reported that hydrophobic amino acids can act on membrane lipid bilayers to reach targets and help scavenge radicals, and histidine significantly enhances antioxidant capacity because the protonation of the imidazole ring acts as a hydrogen donor. Blanco et al. (2017) also suggested that the antioxidant activity of protein hydrolysates is influenced not only by the DH but also by other factors, such as the presence of amino acids that can interact with free radicals, like the SH group in cysteine [65].

The ABTS radical scavenging assay measures the potential of an antioxidant to inhibit the ABTS radical cation. ABTS activity also increased with higher FPH concentrations, with HPHs showing the lowest ABTS activity. However, the EC_50_ values of HPSs and HPHs were not significantly different (Table 4). The ABTS radical scavenging activity of HPSs and HPHs was comparable to those derived from hake by-products using Alcalase [20,66] and to those produced from various fish discards, which had EC_50_ values ranging from 1.12 to 4.93 mg/mL [3]. Protein hydrolysates from common carp roe and blue-spotted stingrays exhibited higher ABTS inhibitory activity, with EC_50_ values of 0.301 mg/mL and 0.79 mg/mL, respectively, compared to those prepared in this study [63,67]. Reported EC_50_ values of hydrolysates prepared from fish discards by-products varied between 3.45 and 22.99 µg/mL [68]. ABTS radical scavenging activity of *Liza abu* muscle protein hydrolysate fractions (<3, 3–10 and 10–30 kDa) varied between 31.79 and 80.11%. Hasani et al. (2022) and Shahosseini et al. (2022) reported that protein hydrolysates prepared from Indian mackerel by-products had an ABTS radical scavenging activity ranging from 31.56 to 91.00% [62,69]. Many studies have reported that a high DH and low MW have a positive correlation with DPPH and ABTS. However, similar to DPPH, it has also been observed that hydrolysates with lower DH exhibit the highest ABTS radical scavenging activity. The antioxidant properties of FPHs are influenced by several factors, including the amino acid composition, chain length (500–1000 Da), sequence of residues/functional side chains, and the methodology used in FPH production [70,71,72]. The elevated levels of methionine, phenylalanine, lysine, leucine, and tyrosine in HPHs may be responsible for enhancing its ABTS radical scavenging activity, similar to its effect on DPPH radical scavenging activity.

The reducing power (RP) assay has also been used to evaluate the potential of any substance to reduce another substance. That can be either by addition or removal of hydrogen or by loss or gain of electrons. To compare the results of the reducing power of FPHs, the A_0.5_ value (the concentration of a sample to produce an absorbance of 0.5) was used (Table 4). A linear increase in the RP with the concentration of hydrolysates was observed for both hydrolysates. This trend has been reported by several authors for protein hydrolysates from fish by-products [20,61,73,74]. The results of this study were comparable in magnitude to those reported by Pires et al. (2013) for Cape hake protein hydrolysates [20]. However, Karoud et al. (2019) reported a higher RP for protein hydrolysates prepared from hake heads, where a 5 mg/mL hydrolysate concentration showed an absorbance of 0.37–0.71 [73]. The A_0.5_ value of FPHs prepared from different discarded species varied between 10 and 31.25 mg/mL, but Henriques et al. (2021) reported A_0.5_ values of 3.19–6.35 mg/mL for FPHs prepared from fish discards and by-products [3,74]. Additionally, Chen et al. (2014) observed a higher RP for protein hydrolysates derived from tilapia sarcoplasmic proteins using papain, where a 3 mg/mL hydrolysate solution exhibited an absorbance of 0.629 ± 0.022. On the contrary, the RP of protein hydrolysates from roe carp (A_0.5_ = 2 mg/mL) reported by Chalamaiah et al. (2012) was lower than that determined in the present work [2,75].

In the present study, strong, significant positive correlations were observed between DH and DPPH (r = 0.989), DH and ABTS (r = 0.971), and DH and RP (r = 0.995). A strong, significant positive correlation was found between Gly and DPPH (r = 0.988), Gly and ABTS (r = 0.963), and Gly and RP (r = 0.981). In contrast, a strong, significant negative correlation was observed between the percentage of peptides with an MW above 1000 Da (MW > 1000 Da) and DPPH (r = −0.927), peptides with an MW > 1000 Da and ABTS (r = −0.986), as well as peptides with an MW > 1000 Da and RP (r = −0.961). In other words, high DH and Gly content result in the highest antioxidant activity. Conversely, a higher percentage of peptides above 1000 Da results in the lowest antioxidant activity.

### 3.7. Fe^2+^ and Cu^2+^ Chelating Activities

Metal-chelating peptides can bind to transition metal ions like Fe^2+^ and Cu^2+^, reducing their activity and functioning as indirect antioxidants. This process helps to reduce or inhibit food oxidation (and consequently, to decrease oxidation products linked to age-related diseases) as well as extend food shelf life. The Fe^2+^ chelating ability of HPSs and HPHs is shown in Table 4. Both hydrolysates presented high Fe^2+^ chelating activity, as evidenced by the lower EC_50_ values achieved. These values were comparable to those referred by Henriques et al. (2021) for hydrolysates prepared from fish discards and by-products [3]. Similarly, studies of fish protein hydrolysates from various Mediterranean fish discards, Cape hake by-products and blue-spotted stingrays reported EC_50_ values of similar magnitudes. The kawakawa fish peptides, particularly those with an MW below 3 kDa, showed EC_50_ values similar to those obtained in this study [66,67,74,76]. On the other hand, lower EC_50_ values were obtained in hydrolysates prepared from starry triggerfish, *Boops boops*, cuttlefish, and smooth-hound and skins and scales of sole fish [77,78,79]. On the contrary, higher EC_50_ values were obtained in hydrolysates prepared from yellow stripe trevally and in tuna liver hydrolysates [64,80].

Several authors reported that the Fe^2+^ chelating capacity increased with the DH [64,81]. However, in the current study, HPSs exhibited the smallest average peptide size and the lowest Fe^2+^ chelating capacity. Notably, there was a strong, significant negative correlation (r = −0.949) between the DH and Fe^2+^ chelating activity. Additionally, a strong, significant positive correlation (r = 0.931) was found between Fe^2+^ chelating activity and the proportion of peptides with an MW greater than 1000 Da.

It has also been described that the chelating capacity of peptides depends not only on their size but also on the composition and sequence of their amino acids. According to Kong and Xiong (2006), the amino acids Asp, Glu, and His are involved in Fe^2+^ chelation, and the aromatic amino acids Phe, Trp, and Tyr also have considerable chelating effects [10]. In fact, in the current study, a strong positive correlation between Fe^2+^ chelating activity and the amino acids Asp (r = 0.953), Glu (r = 0.970), Tyr (r = 0.932), and Phe (r = 0.900) was achieved.

The EC_50_ values of Cu^2+^ chelating activity of HPSs and HPHs are shown in Table 4. Both hydrolysates showed Cu^2+^ chelating activity, but HPHs presented significantly higher activity than HPSs. In the latter, it was not possible to calculate the EC_50_ since 50% inhibition was not reached in the range of concentrations tested. The EC_50_ values of HPSs and HPHs were lower than those found for FPHs of different fish discards and by-products, which varied between 2.49 and 5.66 mg/mL, as reported by Henriques et al. (2021) [3]. The Cu^2+^ chelating capacity of the HPH was also lower than that observed in hydrolysates prepared from Cape hake by-products by Teixeira et al. (2016) (EC_50_ = 1.4 mg/mL) [66]. Low Cu^2+^-chelating ability was also observed by Zhang et al. (2016) for the large yellow croaker peptide fractions corresponding to an MW lower than 3 kDa [82]. However, HPSs showed similar results to those reported by Chai et al. (2015) for hydrolysates obtained from blue-spotted stingray muscle with Alcalase (EC_50_ = 2.14 mg/mL) [66,67,82].

The Cu^2+^ chelating activity of the various protein hydrolysates can be attributed to their high content of carboxylic acids, specifically Asp and Glu [83]. In the present study, a strong positive correlation between Cu^2+^ chelating activity and Asp (r = 0.938) and between Cu^2+^ chelating activity and Glu (r = 0.981) was observed.

Additionally, aromatic amino acids such as Phe and Tyr exhibit significant chelating effects [10], as it is also shown in this work by the strong positive correlations of Tyr vs. Cu^2+^ chelating activity (r = 0.956) and of Phe vs. Cu^2+^ chelating activity (r = 0.981).

The FPHs prepared in this study demonstrated higher chelating activity for Fe^2+^ compared to Cu^2+^, particularly in the case of HPSs.

### 3.8. ACE Inhibitory Activity

The EC_50_ values for ACE inhibitory activity of HPHs and HPSs are shown in Table 4. Both samples inhibited ACE inhibitory activity at the tested concentrations in a concentration-dependent manner, and HPHs presented a significantly higher ACE inhibitory activity, evidenced by the lower EC_50_ value (0.86 ± 0.05 mg/mL). The results obtained in this study were similar to those reported for hydrolysates prepared from Cape hake and salmon by-products with Alcalase [35,37,84]. Henriques et al. (2021) reported relatively lower ACE inhibitory activity for protein hydrolysates from fish discards and by-products (61.20–85.95% for a 5 mg/mL hydrolysate solution) [3]. Fractioned protein hydrolysate from kawakawa was also evaluated for ACE inhibition, and the EC_50_ values ranged from 0.45 to 1.86 mg/mL [76]. Fish protein hydrolysates produced from sardine (EC_50_ = 1.16 mg/mL), tuna industrial by-products (EC_50_ = 0.24–0.27 mg/mL), and Nile tilapia (IC_50_ = 1.2 mg/mL) have shown similar ACE inhibitory activity to HPHs and HPSs [85,86]. However, Abdelhedi et al. (2016) observed relatively low IC_50_ values (75–703 µg/mL) for smooth-hound viscera hydrolysates [87]. It has been reported that chain length, MW, and molecular interaction of the peptides play a crucial role in their antihypertensive activity [88]. Gly and Pro are prevalent amino acids in peptides known for their antihypertensive effects [89,90,91,92]. In this work, strong positive correlations were noted between ACE inhibitory activity and DH (0.946), ACE inhibitory activity and PM < 1000 Da (r = 0.844), and ACE inhibitory activity and Gly (0.921). Additionally, a positive correlation between ACE inhibitory activity and Pro (0.640) was observed. On the contrary, a strong negative correlation was obtained between ACE inhibitory activity and peptides with an MW > 100 Da (r = −0.967).

### 3.9. α-Amylase Inhibitory Activity

**Figure 4:**
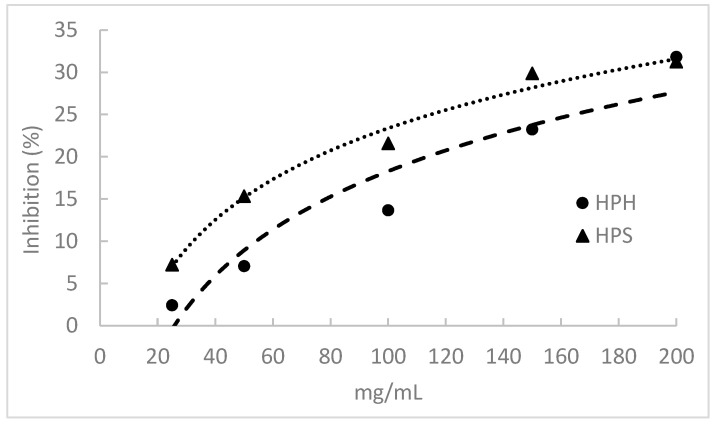
α-Amylase inhibitory activity of protein hydrolysates prepared from salmon heads (HPS) and hake by-products (HPH) by Alcalase.

The α-Amylase inhibitory activity of fish protein hydrolysates is shown in Figure 4. The salmon heads and hake by-products displayed this activity by inhibiting α-amylase in a concentration-dependent manner. However, this activity was relatively low, and none of the hydrolysates caused 50% amylase inhibition at the concentrations tested. HPHs presented significantly lower α-amylase inhibitory activity than HPSs, but at a 200 mg/mL hydrolysate concentration, the inhibition percentage was similar for both hydrolysates (ca. 31%).

The observed results indicate lower inhibition compared to Wan et al. (2023), who reported a 15% inhibition with a 10 mg/mL solution of pompano protein hydrolysates [17]. Henriques et al. (2021) observed major differences in the inhibitory capacity of hydrolysates derived from different fish discards and by-products using Alcalase, with IC_50_ values ranging from 5.70 to 84.37 mg/mL [3]. However, these authors also reported that hydrolysates from whole pouting and red scorpionfish heads did not achieve 50% inhibition within the tested range of concentrations. Similarly, Amini et al. (2017) found α-amylase inhibition for sardinella hydrolysates for a 20 mg/mL solution between 16.61 and 45.71% [18]. The anti-diabetic activity of hydrolysate and peptide fractions obtained from unicorn leatherjacket skins, evaluated by α-amylase inhibition, ranged from 71.17 to 80.45% at a concentration of 1 mg/mL [93]. The IC_50_ values of these collagen peptides were calculated and ranged between 1.17 and 2.65 mg/mL. Slama-Ben et al. (2018) investigated the inhibition of α-amylase using octopus hydrolysates produced with Esperase and *Bacillus subtilis* A26, revealing significantly low IC_50_ values of 61.34 µg/mL and 66.22 µg/mL, respectively [94]. Additionally, Siala et al. (2016) measured the inhibition of α-amylase by grey triggerfish muscle hydrolysates, finding IC_50_ values ranging between 90 and 93 µg/mL [95].

The differences in the inhibitory activity of FPHs may be attributed to the amino acid composition of hydrolysate peptides. Leucine is the most abundant amino acid in peptide sequences associated with antidiabetic properties, as outlined in the review of Farias et al. (2022) [96]. Additionally, Pro and Gly rank as the second and third most frequent amino acids in peptide sequences linked to antidiabetic properties [96]. HPSs had higher levels of Pro and Gly than HPHs, but the latter hydrolysate had higher Leu content. In this study, a negative correlation was found between α-amylase inhibitory activity and DH (r = −0.880). However, a positive correlation was observed between this activity and Leu (r = 0.879) and peptides with an MW > 1000 Da (0.867). The overall analysis of these results suggests that high MW peptides exhibit more efficient inhibition of the α-amylase enzyme.

### 3.10. Salmon Heads Protein Hydrolysates Prepared by SWH

#### 3.10.1. Protein Content, Degree of Hydrolysis, and Hydrolysis Yield

Subcritical Water Hydrolysis (SWH) was employed to prepare salmon head protein hydrolysates, aiming to assess its effectiveness as a sustainable alternative to Alcalase hydrolysis. The protein content of the FPHs prepared by SWH is presented in Table 5. As observed, the protein contents of SWH prepared at 200 °C (SWH1, SWH2, and SWH3) were significantly higher than those prepared at 250 °C (SWH4, SWH5, and SWH6). However, the protein contents of SWH4, SWH5, and SWH6 were very similar to those of HPSs (Table 1). The highest protein content (88.7%) was found at 200 °C, 50 bar, and 30 min (SWH3) and the lowest (70.4%) at 250 °C, 100 bar, and 10 min (SWH5). Different authors have reported that subcritical water temperatures enhance protein solubility due to the increased hydrolysis rate caused by the higher dissociation constant or ion product of subcritical water extraction [6,7]. Noteworthy, the increase in temperature can lead to a greater release of amino acids, promote the degradation of larger amino acids into smaller ones and increase the products of the Maillard reaction or the generation of products such as organic acids [4]. The present results suggest that SWH at 250 °C for shorter times (10 min) promoted the degradation of salmon head proteinaceous material into small products. This result is in line with Hao et al. (2019), who tested a temperature range from 110 °C to 230 °C for 60 min and sufficient pressure to maintain the liquid state to produce abalone (*Haliotis discus hannai* Ino) viscera protein via SWH [6]. These authors found the best response at 170 °C, with extraction yields of 46% and protein concentrations of 60.85%, which are lower than the values found in the present study.

According to Table 5, the DH was significantly (*p* < 0.05) lower for hydrolysates prepared at 200 °C (10.7–11.7%), being higher when SWH was performed at 250 °C (32.4–36.4%). The HPSs exhibited an intermediate DH value (26.2%), higher than those of the SWH at 200 °C but lower than those prepared at 250 °C. The highest DH was achieved with SWH prepared at 250 °C, 100 bar, and 10 min (36.4%). Ahmed et al. (2018) and Lee et al. (2018) also obtained a higher DH at a higher tested temperature (250 °C) to produce tuna skin and Pacific oyster hydrolysates, respectively [21].

It was found that hydrolysis yield decreased with the increase in temperature (Table 5). The highest hydrolysis yield in salmon hydrolysates was 94.3% at 200 °C, 50 bar, and 30 min. On the contrary, the lowest value (47.0%) was obtained with hydrolysates prepared by SWH at 250 °C, 100 bar, and 10 min. The hydrolysates prepared by SWH presented higher hydrolysis yield value than the hydrolysates prepared using Alcalase, apart from the one prepared at 250 °C, 100 bar, and 10 min.

Several authors reported that increased temperature enhances hydrolysis efficiency, resulting in greater release of amino acids/peptides [4,21,97]. This phenomenon can be attributed to the higher ionization constant of water. However, this contrasts with the findings observed in this study [98,99,100].

#### 3.10.2. Molecular Weight

**Figure 5:**
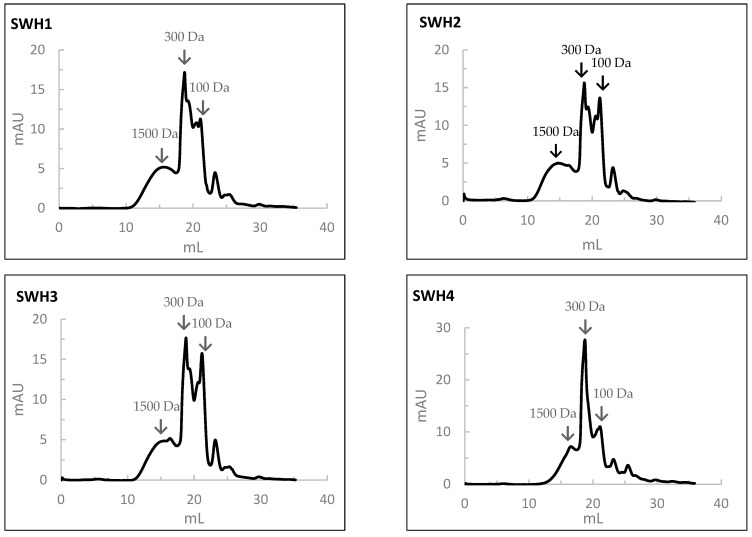

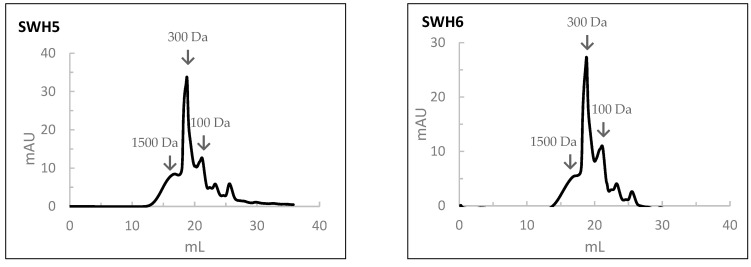
The molecular weight distribution of peptides obtained by size-exclusion chromatography analysis of salmon head hydrolysates prepared by Subcritical Water Hydrolysis under the different conditions: SWH1—200 °C, 100 bar, 30 min); SWH2—200 °C, 100 bar, 10 min; SWH3—200 °C, 50 bar, 30 min; SWH4—250 °C, 100 bar, 30 min; SWH5—250 °C, 100 bar, 10 min; SWH6—250 °C, 50 bar, 30 min.

The results of the MW profile of SWH are shown in Figure 5. In the SWH at 200 °C, the percentages in the average MW of peptides of 1500, 300, 100, and <100 Da were 30%, 35%, 27%, and 8%, respectively. With the increase in the subcritical water temperature, the percentage of peptides with a high MW decreased while that of a low molecular weight increased. In this case, the percentages of the average MW of peptides of 1500, 300, 100, and <100 Da were 20%, 45%, 20%, and 5%, respectively.

When comparing the MW distribution of hydrolysates prepared by SWH to those prepared by HPSs, the percentage of peptides with an average MW of 100 Da was relatively higher in the HPSs.

#### 3.10.3. Antioxidant Activity

During SWH, several low-molecular-weight peptides and free amino acids were generated, depending on temperature and time. These compounds are believed to be responsible for the free-radical scavenging activities and reducing the power of the hydrolysates [98,101].

In this work, the DPPH radical scavenging activity of SWH prepared at 250 °C was generally higher than that of SWH prepared at 200 °C (Table 6). Moreover, for the same temperature and pressure, the DPPH radical scavenging activity of hydrolysates prepared for 30 min was not significantly different from that of the hydrolysates prepared for 10 min. The highest DPPH activity was observed for SWH4 prepared at 250 °C, 100 bar, and 30 min (22.9%). On the contrary, the lowest value (9.6%) was achieved when SWH was performed at 200 °C, 50 bar, and 30 min. Overall, SWH presented significantly (*p* < 0.05) higher DPPH radical scavenging than HPSs.

Although several authors have noted that DPPH radical scavenging activity increases with increasing hydrolysis temperature, the literature remains inconclusive [7,98,99,102,103]. For example, Asaduzzaman et al. (2015) noted increased DPPH radical scavenging activity at 220 °C during SWH, whereas higher temperatures of 250 °C to 280 °C led to decreased activity [100]. Abalone viscera subcritical water hydrolysates prepared at 230 °C had also lower radical scavenging activity than those prepared at 200 °C [6]. Accordingly, Lee et al. (2018) reported that Pacific oyster hydrolysates prepared by SWH at 250 °C and 120 bar had lower DPPH radical scavenging activity than hydrolysates prepared at 200 °C and 80 bar [21].

In contrast to HPSs (5.45%), SWH exhibited relatively lower ABTS radical scavenging activity at the tested concentrations, ranging from 1.89% (SWH3 prepared at 200 °C, 50 bar, 30 min) to 3.10% (SWH4 prepared at 250 °C, 100 bar, 30 min), as depicted in Table 6. In general, a similar trend was observed for DPPH and ABTS radical scavenging activities among the SWH, i.e., hydrolysates prepared at 250 °C tended to exhibit higher values of these activities. Moreover, at the same temperature and pressure, there was no significant difference in the ABTS radical scavenging activity between hydrolysates prepared for 30 min and those prepared for 10 min.

Several authors had reported increased ABTS radical scavenging activity of subcritical water hydrolysates with increasing temperature [5,21,98,102,103]. By contrast, no differences were observed in the ABTS radical scavenging activity of squid muscle hydrolysates prepared at different temperatures (160–280 °C). Likewise, Lee et al. (2018) reported that Pacific oyster hydrolysates prepared at 250 °C had lower ABTS radical scavenging activity than hydrolysates prepared at 200 °C [21].

Regarding the RP results of SWH, no significant differences were observed in the RP of different hydrolysates (Table 6). Furthermore, the results obtained with SWH were not significantly different from those of HPSs. Different results were observed by Asaduzzaman et al. (2020), who found that skin and bone hydrolysates prepared by SWH at 250 °C exhibited a higher RP (absorbance at 5 mg/mL was approximately 0.5 and 0.65, respectively) than those prepared at 200 °C (absorbance at 5 mg/mL was approximately 0.42 and 0.5, respectively) [103]. Asaduzzaman et al. (2015) studied the RP of squid muscle hydrolysates prepared by SWH at different temperatures (160–280 °C) and observed that those prepared at 220 °C showed the highest value (Abs = 1.45) [100].

Pressure is a crucial parameter that can influence the yields and bioactivities of salmon head protein hydrolysates produced by SWH. In this study, two pressure conditions (50 and 100 bar) were assessed while keeping the reaction time (30 min) and temperature (200 or 250 °C) constant. Data from Table 5 indicate that pressure did not affect the protein content and degree of hydrolysis (DH%) of salmon head protein isolates, as there were no statistical differences between SWH1 (200 °C, 100 bar, 30 min) and SWH3 (200 °C, 50 bar, 30 min), nor between SWH4 (250 °C, 100 bar, 30 min) and SWH6 (250 °C, 50 bar, 30 min). However, Table 6 shows that for the same temperature and time conditions (SWH1 versus SWH3 and SWH4 versus SWH6), hydrolysates prepared at 100 bar had significantly higher (*p* < 0.05) antioxidant activities, including DPPH and ABTS radical scavenging activities and Fe2+ chelating activity. The SWH pressure (50 or 100 bar) did not affect the reducing power of the hydrolysates. Ahmed and Chun (2018) compared hydrolysates from tuna skin and isolated tuna skin collagen using the SWH process at 150–300 °C and 50–100 bar, reporting that hydrolysates prepared at 250 °C and 50 bar for 5 min showed significantly (*p* < 0.05) higher antioxidant activity (measured by ABTS, DPPH, FRAP, and metal chelating activity) than those prepared at lower temperatures of 150 ºC and 180 °C. As noted by Rivas-Vela et al. (2021) [3], the combined effects of SWH pressure and temperature, rather than considering these factors separately, significantly influence protein hydrolysis to produce small peptides and amino acids.

## 4. Conclusions

In the present study, fish protein hydrolysates were prepared from salmon heads and Cape hake trimmings using Alcalase. Furthermore, subcritical water hydrolysis was also used to produce hydrolysates from salmon heads. The gel filtration profile of different hydrolysates indicates that proteins were hydrolysed into peptides with smaller molecular weight, which was according to the DH achieved. All hydrolysates were a valuable resource for protein, amino acids (except for histidine), and minerals. Moreover, they contained significantly lower Pb and Hg levels than those regulated. The cytotoxicity results allow the conclusion that increasing the concentration of hydrolysates is safe and does not compromise cell metabolism. Both fish hydrolysates also promoted cell growth. Regarding antioxidant and chelating activities, HPHs presented higher biological activities. HPSs had higher α-amylase inhibitory activity, but HPHs showed significantly higher ACE inhibitory activity.

Comparing enzymatic hydrolysates and those prepared by SWH, the results indicated that SWH at 250 °C could enhance the DPPH radical scavenging activity and chelating properties of the peptides obtained. These findings suggest that protein hydrolysates prepared in this study, especially those prepared with Cape hake trimmings, are a promising natural source of nutritional compounds and bioactive peptides that make them potential candidates for use as ingredients in new food products or nutraceuticals, while at the same time reducing fish waste. It can also be concluded that SWH is a viable alternative to enzymatic methods to produce FPHs from salmon heads with high antioxidant and chelating properties.

## Figures and Tables

**Table 1:** Proximate composition of salmon heads and hake by-products (ww) and protein hydrolysates prepared from salmon heads (HPS, dw) and hake by-products (HPH, dw) with Alcalase, and degree of hydrolysis (DH) and protein yield of the hydrolysis process.

	Protein (%)	Moisture (%)	Ash (%)	Fat (%)	DH (%)	Protein Yield (%)	Hydrolysis Yield (%)
Salmon heads	13.8 ± 0.9 ^A^	61.2 ± 0.5 ^A^	2.39 ± 0.19 ^A^	19.8 ± 0.2 ^B^	---	---	
Hake by-products	14.3 ± 1.4 ^A^	81.6 ± 0.0 ^B^	2.76 ± 0.13 ^B^	0.5 ± 0.0 ^A^	---	---	
HPS	75.6 ± 0.9 ^a^	7.6 ± 0.5 ^b^	13.45 ± 0.31 ^a^	2.1 ± 0.1 ^a^	26.2 ± 0.2 ^b^	68.1 ± 2.3 ^a^	32.4 ± 2.3 ^a^
HPH	76.8 ± 0.5 ^a^	4.4 ± 0.1 ^a^	16.69 ± 0.16 ^b^	2.7 ± 0.0 ^b^	20.3 ± 0.0 ^a^	75.5 ± 3.4 ^b^	76.4 ± 3.4 ^b^

Different lowercase (a, b) letters in each column indicate significant differences (*p* < 0.05) between FPH. Different uppercase (A, B) letters in each column indicate significant differences (*p* < 0.05) between raw materials.

**Table 2:** Essential (EAA) and non-essential (NEAA) amino acids expressed in g/100 g dw sample (g/100 g protein) of hake by-products, salmon heads, and protein hydrolysates prepared from hake by-products (HPH) and salmon heads (HPS) by Alcalase hydrolysis.

EAA	Hake By-Products	Salmon Heads	HPH	HPS
HIS	1.33 ± 0.10 ^b^ (1.70 ± 0.13) ^A^	0.58 ± 0.10 ^a^ (1.63 ± 0.27) ^A^	0.75 ± 0.15 ^a,b^ (0.97 ± 0.20) ^A^	1.06 ± 0.37 ^a,b^ (1.40 ± 0.48) ^A^
LYS	5.68 ± 1.67 ^a^ (7.30 ± 2.15) ^A^	3.38 ± 0.46 ^a^ (9.51 ± 1.30) ^A^	6.24 ± 0.61 ^a^ (8.12 ± 0.80) ^A^	6.09 ± 0.92 ^a^ (8.05 ± 1.22) ^A^
LEU	5.95 ± 0.05 ^d^ (7.64 ± 0.06) ^A^	2.22 ± 0.25 ^a^ (6.25 ± 0.70) ^B^	5.42 ± 0.11 ^c^ (7.05 ± 0.14) ^A,B^	4.60 ± 0.02 ^b^ (6.08 ± 0.02) ^B^
VAL	3.75 ± 0.22 ^c^ (4.81 ± 0.28) ^A^	1.53 ± 0.12 ^a^ (4.30 ± 0.33) ^A^	3.43 ± 0.03 ^b,c^ (4.46 ± 0.04) ^A^	3.13 ± 0.03 ^b^ (4.13 ± 0.04) ^A^
MET	1.90 ± 0.50 ^b^ (2.44 ± 0.64) ^A^	0.47 ± 0.08 ^a^ (1.31 ± 0.21) ^A^	1.56 ± 0.08 ^b^ (2.02 ± 0.10) ^A^	1.44 ± 0.11 ^b^ (1.91 ± 0.15) ^A^
CYS	0.79 ± 0.07 ^b^ (1.01 ± 0.08) ^B^	0.51 ± 0.06 ^a^ (1.44 ± 1.18) ^A^	0.89 ± 0.03 ^b^ (1.16 ± 0.04) ^A,B^	1.05 ± 0.01 ^c^ (1.39 ± 0.02) ^A^
PHE	3.13 ± 0.17 ^c^ (4.02 ± 0.21) ^A^	1.21 ± 0.13 ^a^ (3.39 ± 0.36) ^A,B^	2.89 ± 0.06 ^c^ (3.76 ± 0.07) ^A,B^	2.47 ± 0.06 ^b^ (3.27 ± 0.08) ^B^
TYR	2.69 ± 0.16 ^c^ (3.45 ± 0.21) ^A^	1.05 ± 0.10 ^a^ (2.94 ± 0.29) ^A,B^	2.42 ± 0.04 ^c^ (3.15 ± 0.05) ^A,B^	2.06 ± 0.06 ^b^ (2.73 ± 0.08) ^B^
ILE	3.40 ± 0.24 ^c^ (4.37 ± 0.30) ^A^	1.28 ± 0.12 ^a^ (3.58 ± 0.34) ^B^	2.93 ± 0.07 ^b^ (3.81 ± 0.10) ^A,B^	2.60 ± 0.02 ^b^ (3.44 ± 0.03) ^B^
THR	4.03 ± 0.24 ^b^ (5.18 ± 0.31) ^A^	1.93 ± 0.20 ^a^ (5.41 ± 0.57) ^A^	4.02 ± 0.01 ^b^ (5.23 ± 0.01) ^A^	3.53 ± 0.13 ^b^ (4.67 ± 0.18) ^A^
NEAA				
ASP	8.16 ± 0.33 ^c^ (10.48 ± 0.42) ^A^	3.22 ± 0.34 ^a^ (9.05 ± 0.95) ^A^	7.57 ± 0.15 ^b,c^ (9.86 ± 0.20) ^A^	6.80 ± 0.09 ^b^ (8.98 ± 0.12) ^A^
GLU	12.96 ± 0.55 ^c^ (16.64 ± 0.70) ^A^	4.79 ± 0.50 ^a^ (13.44 ± 1.40) ^B^	12.11 ± 0.22 ^c^ (15.76 ± 0.28) ^A,B^	10.12 ± 0.12 ^b^ (13.38 ± 0.17) ^B^
ASN	<QL (<QL)	<QL (<QL)	<QL (<QL)	<QL (<QL)
SER	3.52 ± 0.15 ^b^ (4.52 ± 0.10) ^A^	1.49 ± 0.19 ^a^ (4.17 ± 0.54) ^A^	3.50 ± 0.07 ^b^ (4.55 ± 0.09) ^A^	3.14 ± 0.10 ^b^ (4.15 ± 0.13) ^A^
GLN	<QL (<QL)	<QL (<QL)	<QL (<QL)	<QL (<QL)
GLY	4.14 ± 0.05 ^a^ (5.31 ± 0.07) ^C^	3.27 ± 0.53 ^a^ (9.17 ± 1.49) ^A,B^	5.47 ± 0.12 ^b^ (7.12 ± 0.16) ^B,C^	7.31 ± 0.15 ^c^ (9.66 ± 0.20) ^A^
ARG	4.86 ± 0.16 ^b^ (6.24 ± 0.20) ^A^	2.21 ± 0.26 ^a^ (6.22 ± 0.74) ^A^	4.74 ± 0.12 ^b^ (6.17 ± 0.15) ^A^	4.58 ± 0.14 ^b^ (6.05 ± 0.19) ^A^
ALA	4.98 ± 0.19 ^b^ (6.39 ± 0.25) ^A^	2.42 ± 0.25 ^a^ (6.80 ± 0.70) ^A^	5.16 ± 0.03 ^b^ (6.72 ± 0.04) ^A^	5.25 ± 0.11 ^b^ (6.94 ± 0.15) ^A^
Tau	<QL (<QL)	<QL (<QL)	<QL (<QL)	<QL (<QL)
PRO	3.11 ± 0.41 ^b^ (3.99 ± 0.53) ^A^	1.80 ± 0.22 ^a^ (5.05 ± 0.61) ^A^	3.49 ± 0.10 ^b^ (4.54 ± 0.13) ^A^	3.91 ± 0.36 ^b^ (5.17 ± 0.48) ^A^
HYP	<QL (<QL)	0.42 ± 0.07 ^a^ (1.19 ± 0.19) ^A^	<QL (<QL)	1.00 ± 0.03 ^b^ (1.32 ± 0.04) ^A^
Total AA (g/100 g)	74.38	33.77	72.57	70.14

Different letters in each line indicate significant differences (*p* < 0.05) for each amino acid. QL—quantification limit.

**Table 3:** Minerals (mg/kg) and contaminant metals (mg/kg) of Cape hake by-products (dw), salmon heads (dw), and protein hydrolysates (dw) prepared from hake by-products (HPHs) and salmon heads (HPSs) and Dietary Reference Intake (DRI, mg/day, %) of hake by-products (HPHs) and salmon heads (HPS).

Minerals (mg/kg)	DRI (mg/Day)	Cape Hake By-Products	Salmon Heads	HPH		HPS	
				mg/kg	DRI (%)	mg/kg	DRI (%)
Na	1500	6985± 1349 ^a^	5425 ± 582 ^a^	58,075 ± 3722 ^b^	387.2	49,295 ± 1493 ^b^	328.6
K	4700	53,845 ± 1008 ^a^	10,721 ± 1405 ^b^	15,315 ± 622 ^c^	32.6	12,107 ± 104 ^b^	25.8
Mg	420	1657 ± 104 ^c^	1445 ± 317 ^c^	335 ± 8 ^b^	8.0	29 ± 2 ^a^	0.7
Fe	18	13.8 ± 3.4 ^b^	22.9 ± 1.6 ^c^	3.0 ± 0.1 ^a^	1.7	18.0 ± 0.1 ^b,c^	10.0
Zn	11	9.7 ± 0.1 ^b^	24.2 ± 2.1 ^c^	3.1 ± 0.0 ^a^	2.8	7.0 ± 0.1 ^a,b^	6.4
Mn ^#^	-	2.7 ± 0.4 ^a^	8.7 ± 0.9 ^b^	<DL	-	<DL	-
Cr ^##^	-	0.576 ± 0.08 ^a^	0.713 ± 0.07 ^a^	<DL	-	<DL	-
Ni	-	0.056 ± 0.002 ^a^	0.113 ± 0.011 ^a^	0.111 ± 0.002 ^a^	-	0.154 ± 0.07 ^a^	-
Cu	0.9	0.838 ± 0.05 ^a^	2.25 ± 0.35 ^b^	0.57 ± 0.01 ^a^	6.3	0.53 ± 0.01 ^a^	5.9
Contaminant metals (mg/kg)							
Pb *		0.002 (<DL)	0.002 (<DL)	0.04 (<QL)		0.07 ± 0.00	
Cd **		0.003 (<QL)	0.001 (<DL)	0.004 (<QL)		0.002 (DL)	
Hg ***		0.43 ± 0.16	0.009 (<QL)	0.18 ± 0.00		0.013 ± 0.000	

Different letters indicate significant differences between samples within each metal. DRI—recommended daily values used in the calculation of % DRI. DL—detection limit; QL—quantification limit; ^#^ DL = 0.01 mg/kg; ^##^ DL = 0.09 mg/kg; * DL = 0.02 mg/kg; QL = 0.06 mg/kg; ** DL = 0.002 mg/kg; QL = 0.006 mg/kg; *** DL = 0.004 mg/kg.

**Table 4:** ABTS and DPPH radical scavenging activities, reducing power (RP), Fe^2+^ and Cu^2+^ chelating activities, and ACE inhibitory activity of protein hydrolysates prepared from salmon heads (HPSs) and hake by-products (HPHs).

Sample	EC_50_/A_0.5_ * (mg/mL)					
	ABTS	DPPH	RP *	Fe^2+^	Cu^2+^	ACE
HPS	2.4 ± 0.01 ^a^	10.5 ± 0.04 ^b^	25.5 ± 0.16 ^b^	0.45 ± 0.04 ^a^	n.a.	2.2 ± 0.13 ^b^
HPH	2.1 ± 0.05 ^a^	9.5 ± 0.10 ^a^	20.1 ± 0.35 ^a^	0.52 ± 0.02 ^b^	0.64 ± 0.01	0.86 ± 0.05 ^a^

n.a.—not achieved—50% inhibition was not achieved in the range of concentrations tested. * In the case of reducing power, A_0.5_ was determined. Different letters in each column indicate significant differences (*p* < 0.05) between FPH.

**Table 5:** Protein content (P), degree of hydrolysis (DH), and hydrolysis yield of salmon heads hydrolysates prepared by Subcritical Water Hydrolysis (SWH).

FPH	P (%)	DH (%)	Hydrolysis Yield (%)
SWH1 (200 °C, 100 bar, 30 min)	84.8 ± 0.5 ^c^	10.7 ± 0.13 ^a^	83.5 ± 0.53 ^d^
SWH2 (200 °C, 100 bar, 10 min)	86.2 ± 0.8 ^c^	11.6 ± 0.02 ^a^	93.3 ± 0.78 ^e^
SWH3 (200 °C, 50 bar, 30 min)	88.7 ± 0.6 ^c^	11.7 ± 0.29 ^a^	94.3 ± 0.42 ^e^
SWH4 (250 °C, 100 bar, 30 min)	75.8 ± 0.4 ^b^	33.8 ± 1.02 ^b^	70.3 ± 0.23 ^c^
SWH5 (250 °C, 100 bar, 10 min)	70.4 ± 0.4 ^a^	36.4 ± 0.62 ^c^	47.0 ± 0.21 ^a^
SWH6 (250 °C, 50 bar, 30 min)	76.8 ± 0.6 ^b^	32.4 ± 0.15 ^b^	63.8 ± 0.08 ^b^

Different letters in each column indicate significant differences (*p* < 0.05) between fish protein hydrolysates.

**Table 6:** DPPH and ABTS radical scavenging activities, reducing power (RP), Cu^2+^ and Fe^2+^ chelating activities of salmon heads hydrolysates (0.1 mg/mL) prepared by Subcritical Water Hydrolysis and by Alcalase hydrolysis.

	Antioxidant Activity (%)			Chelating Activity (%)	
	DPPH	ABTS	RP	Cu^2+^	Fe^2+^
SWH1 (200 °C, 100 bar, 30 min)	17.9 ± 0.53 ^c^	2.50 ± 0.17 ^a,b^	0.061 ± 0.006 ^a^	9.7 ± 0.4 ^a^	1.7 ± 0.40 ^a^
SWH2 (200 °C, 100 bar, 10 min)	15.2 ± 0.83 ^c^	2.40 ± 0.13 ^a,b^	0.058 ± 0.000 ^a^	12.8 ± 2.7 ^a,b^	0.37 ± 0.08 ^a^
SWH3 (200 °C, 50 bar, 30 min)	9.6 ± 0.09 ^b^	1.89 ± 0.63 ^a^	0.061 ± 0.000 ^a^	12.0 ± 1.7 ^a,b^	1.4 ± 0.40 ^a^
SWH4 (250 °C, 100 bar, 30 min)	22.9 ± 0.28 ^d^	3.10 ± 0.07 ^b^	0.064 ± 0.002 ^a^	11.2 ± 1.1 ^a^	13.0 ± 0.8 ^d^
SWH5 (250 °C, 100 bar, 10 min)	18.8 ± 0.80 ^c,d^	2.78 ± 0.17 ^a,b^	0.054 ± 0.009 ^a^	13.0 ± 0.4 ^a,b^	6.5 ± 0.8 ^c^
SWH6 (250 °C, 0 bar, 30 min)	14.9 ± 2.2 ^c^	2.87 ± 0.01 ^a,b^	0.058 ± 0.001 ^a^	16.7 ± 1.7 ^c^	2.5 ± 0.10 ^a,b^
HPS (60 °C, pH 8.5, 3 h, 1% Alcalase)	0.83 ± 0.08 ^a^	5.45 ± 0.48 ^c^	0.051 ± 0.002 ^a^	nd	3.1 ± 0.62 ^a,b^

nd—not detected at 0.1 mg/mL hydrolysate solution; Different letters in each column indicate significant differences (*p* < 0.05) between FPH.

## Data Availability

The original contributions presented in the study are included in the article/supplementary material, further inquiries can be directed to the corresponding author.

## Supplementary Materials

The following supporting information can be downloaded at: https://www.mdpi.com/article/10.3390/foods13152418/s1, Figure S1: Study Design on Protein Hydrolysates from Salmon Heads and Cape Hake By-Products; Table S1: Different combinations of temperature, pressure and time tested in the subcritical water hydrolysis (SWH). Table S2: Amino acids score (%) of protein hydrolysates prepared from hake by-products (HPH) and salmon heads (HPS) by Alcalase hydrolysis.
