# Supplementary material for: Protein Hydrolysates from Salmon Heads and Cape Hake By-Products: Comparing Enzymatic Method with Subcritical Water Extraction on Bioactivity Properties
Source: Foods. 2024 Jul 30;13(15):2418. doi: 10.3390/foods13152418 (PMC11311982; doi:10.3390/foods13152418)
Supplement: Supplementary file 1 [file foods-13-02418-s001.zip › foods-3123024-supplementary.pdf]

Supplementary material

Table S1 – Different combinations of temperature (T), pressure (P) and time tested in the subcritical water hydrolysis (SWH).

	T (ºC)	P (bar)	Time (min)
SWH1	200	100	30
SWH2	200	100	10
SWH3	200	50	30
SWH4	250	100	30
SWH5	250	100	10
SWH6	250	50	30

Table S2 - Amino acids score (%) of protein hydrolysates prepared from hake by-products (HPH) and salmon heads (HPS) by Alcalase hydrolysis.

	**FAO/WHO standard*** (mg/g of protein)	**HPH – amino acids score**	**HPS – amino acids score**
**THR**	23	227.5 ± 0.5	203.2 ± 9.3
**CYS**	6	192.7 ± 7.3	232.0 ± 3.8
**LYS**	45	180.5 ± 21.8	178.9 ± 33.2
**PHE+TYR**	38	181.7 ± 3.8	157.8 ± 5.2
**ILE**	30	127.1 ± 3.9	114.5 ± 1.2
**MET**	16	126.5 ± 8.0	119.2 ± 11.2
**LEU**	59	119.5 ± 2.9	103.0 ± 0.5
**VAL**	39	114.4 ± 1.1	106.0 ± 1.4
**HIS**	15	64.9 ± 16.0	93.4 ± 39.5

* FAO/WHO/UNU, 2007

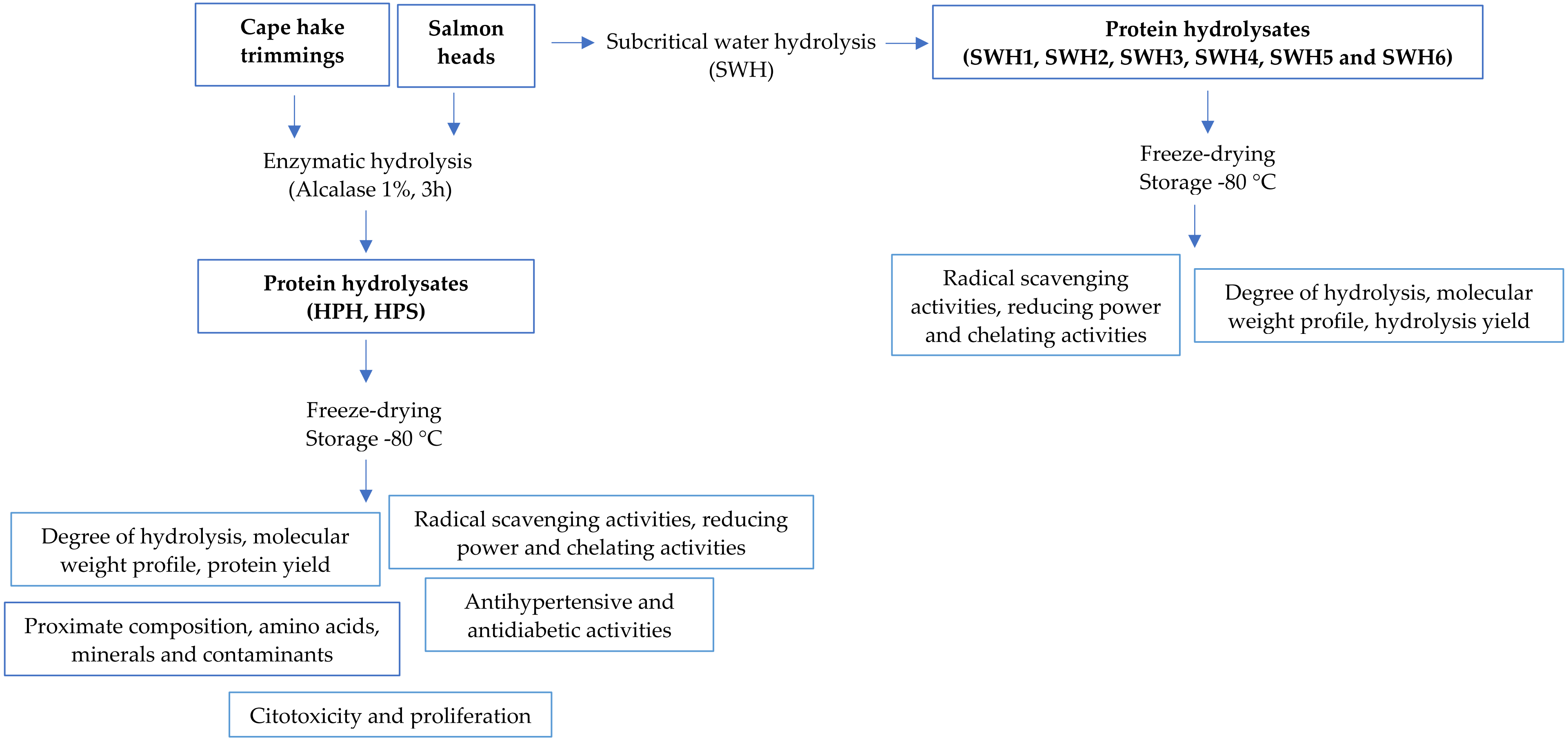

Figure S1 – Study Design on Protein Hydrolysates from Salmon Heads and Cape Hake By-Products.
